# Oxidative stress, autophagy and pyroptosis in the neovascularization of oxygen-induced retinopathy in mice

**DOI:** 10.3892/mmr.2020.11753

**Published:** 2020-12-03

**Authors:** Shuai Wang, Li-Yang Ji, Li Li, Jing-Min Li

Mol Med Rep 19: 927-934, 2019; DOI: 10.3892/mmr.2018.9759

Subsequently to the publication of this paper, the authors have realized that Figs. 2 and 5 have been published containing the same GAPDH control protein bands. After having examined the final proofs of this article, the control blots were indeed different comparing between the figures, and regrettably an error concerning Fig. 2 was made during the final stages of the proof preparation.

The corrected version of Fig. 2, including the correct GAPDH protein bands, is shown opposite. Note that the error that occurred with this Figure during production process did not affect the results or the conclusions reported in this paper, and all the authors agree to this Corrigendum. The Editor of *Molecular Medicine Reports* apologizes to the authors and to the readership for any inconvenience caused.

## Figures and Tables

**Figure 2.**
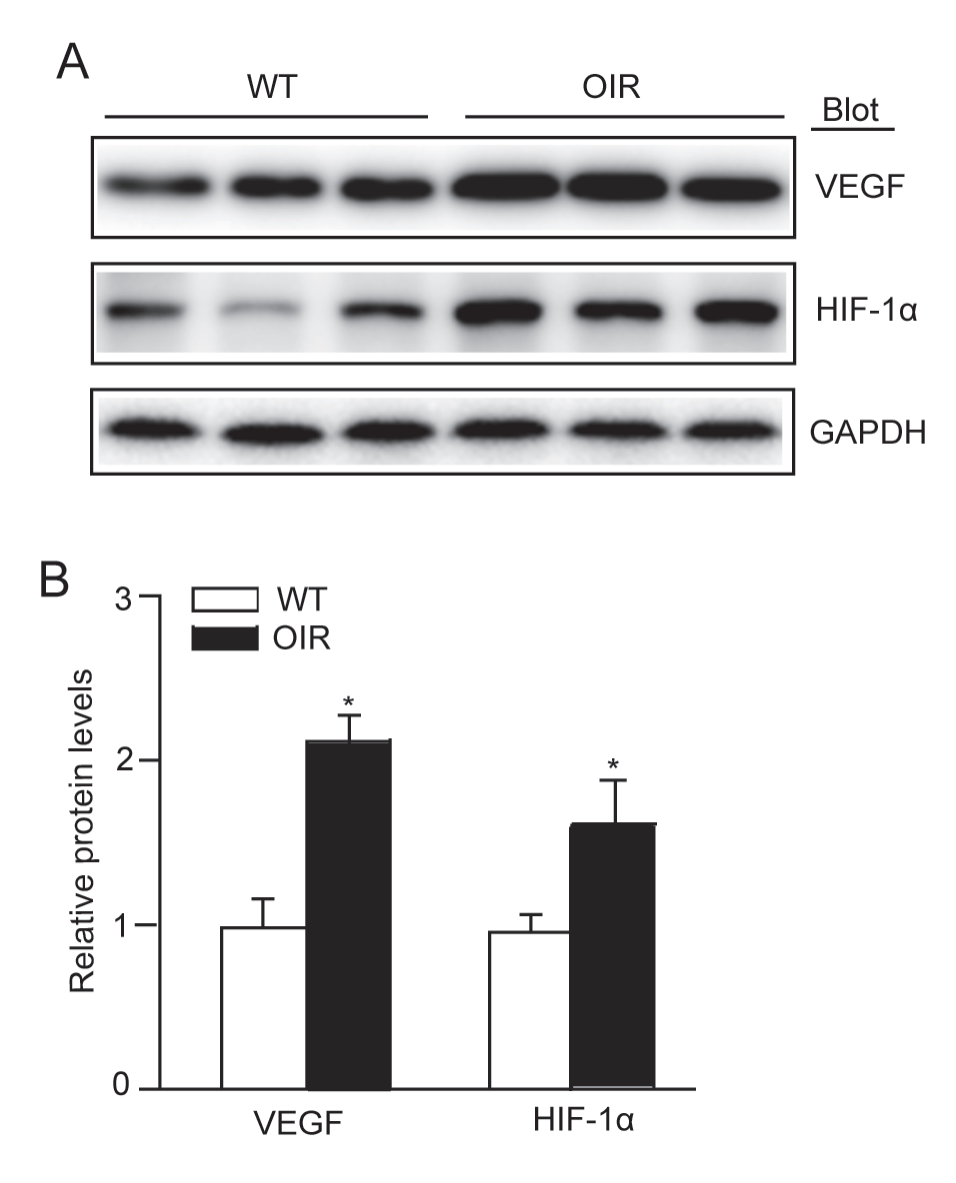
VEGF-A expression is activated in the retinas of OIR mice. (A) Immunoblot analysis of the protein expression levels of VEGF-A and HIF-1α in the retinas. (B) Quantification revealed an increase in the expression levels of VEGF-A and HIF-1α in the retinas of the OIR mice compared with WT mice. The relative protein expression level was normalized to GAPDH (n=3 mice per group). Data are presented as the mean ± standard deviation of the mean. *P<0.05 vs. WT mice. OIR, oxygen-induced retinopathy; WT, wild-type; VEGF, vascular endothelial growth factor; HIF-1α, hypoxia inducible factor-1α.

